# Infection of Raccoon Dogs (*Nyctereutes procyonoides*) from Northern Poland with Gastrointestinal Parasites as a Potential Threat to Human Health

**DOI:** 10.3390/jcm11051277

**Published:** 2022-02-26

**Authors:** Bogumiła M. Pilarczyk, Agnieszka K. Tomza-Marciniak, Renata Pilarczyk, Izabella Rząd, Małgorzata J. Bąkowska, Jan M. Udała, Agnieszka Tylkowska, Viktoriia Havryliak

**Affiliations:** 1 Department of Animal Reproduction Biotechnology and Environmental Hygiene, Faculty of Biotechnology and Animal Husbandry, West Pomeranian University of Technology, Szczecin, 71-270 Szczecin, Poland; bogumila.pilarczyk@zut.edu.pl (B.M.P.); malgorzata.bakowska@zut.edu.pl (M.J.B.); jan.udala@zut.edu.pl (J.M.U.); 2 Laboratory of Biostatistics, Faculty of Biotechnology and Animal Husbandry, West Pomeranian University of Technology, Szczecin, 71-270 Szczecin, Poland; renata.pilarczyk@zut.edu.pl; 3 Institute of Marine and Environmental Sciences, University of Szczecin, 71-415 Szczecin, Poland; izabella.rzad@usz.edu.pl; 4 Department of Animal Environment Biology, Faculty of Animal Breeding, Bioengineering and Conservation, Warsaw University of Life Sciences, 02-787 Warsaw, Poland; agnieszka_tylkowska@sggw.edu.pl; 5 Department of Technology of Biologically Active Substances, Pharmacy and Biotechnology, Institute of Chemistry and Chemical Technologies, Lviv Polytechnic National University, 79000 Lviv, Ukraine; vitahavryliak@gmail.com

**Keywords:** raccoon dog, parasitic zoonoses, *Echinococcus multilocularis*, *Toxocara canis*, *Alaria alata*, Poland

## Abstract

The aim of the study was to determinate the prevalence and intensity of infection of raccoon dogs with internal parasites, with a particular emphasis on particular species of helminths known to be dangerous to humans. A total of 96 raccoon dogs were obtained from hunters from September 2018 to October 2021. The digestive tract was taken for examination. The parasitological examination was performed using the dissection methods. The extensity of infection with all internal parasites was 60.3%. The following parasites were found in the tested animals: *Echinococcus multilocularis* (in 10.42% of animals), *Toxocara canis* (18.75%), *Alaria alata* (25.0%), *Taenia* spp. (19.79%), *Uncinaria stenocephala* (27.08%), *Mesocestoides* spp. (54.17%) and *Dipylidium caninum* (6.25%). The highest mean intensity of infection was demonstrated by *A. alata* and *E. multilocularis* then by *Mesocestoides* spp. This study showed that the raccoon dog from northern Poland is a reservoir host of zoonotic pathogens, such as *E. multilocularis*, *Toxocara canis* and *Alaria alata*. Although the role of the racoon dog as a final host of the life cycle of *E. multilocularis* is considered of less importance than that of the red fox, this species may increase the risk of echinococcosis in humans, mainly due to its growing population in northern Poland.

## 1. Introduction

The raccoon dog (*Nyctereutes procyonoides*) is one of the most widely-distributed invasive mammal species in Europe [1], with a population density ranging from 1 to 5 individuals per km^2^ [2,3]. In Poland, a region with favourable climatic conditions, their numbers continue to grow, as does their territorial range [4]. As the animals have recently moved from the forest environment to cultivated fields, being observed in the vicinity of villages [1], their role as a vector of zoonoses may gain prominence. 

It was found that raccoon dogs can be infected with a minimum of 32 helminth species, of which 19 are zoonotic [5]. The most significant pathogens transmitted by raccoon dogs are arguably the tapeworm *Echinococcus multilocularis* and *Toxocara canis*. The larval forms of *E. multilocularis* are known to cause alveococcosis, a very dangerous zoonotic disease. Its development in humans occurs over a long period of time and may last from 5 to 15 years. As such, the disease is most often diagnosed late, and if untreated, usually ends in death [6,7]. It is hence considered the most dangerous zoonosis present in the temperate climate of Europe. 

In 2019, EU Member States reported 752 laboratory-confirmed cases of echinococcosis, with a notification rate of 0.18 cases per 100,000 residents. The highest notification rate was recorded in Lithuania (2.90 cases per 100,000 residents), followed by Bulgaria (2.76) and Austria (0.41). In Poland the notification rate was 0.18, with 70 cases reported in total. However, due to its long incubation period and non-specific symptoms, it is difficult to estimate the true burden of alveolar echinococcosis [8].

The invasive eggs of *E. multilocularis* are spread through the environment with the faeces of the final hosts, these being foxes and raccoon dogs in Europe, and these represent the primary source of infection for humans. Therefore, greater monitoring is needed to assess the epidemiological situation, i.e., about the prevalence and intensity of infection, in these animal species. The resulting information can provide insight into the risk of infection for humans. 

Toxocarosis, caused by *Toxocara canis*, is one of the most common zoonoses globally, and in Western Europe, 2–5% of adults from urban areas and 14.2–37% of adults in rural areas have been found to be seropositive [9]. In Poland, between 9 and 249 clinical cases of toxocarosis were reported annually in the years 2002–2009 [10]. Recommendations on how to reduce the incidence of toxocariasis in humans mainly focus on deworming pets, typically dogs, disposing of their faeces and following hygiene procedures. However, little attention is paid to the fact that free-living animals such as raccoon dogs or foxes may act as sources of invasive larvae in the environment. 

Another zoonotic parasitosis is alariosis, caused by infection with the larval stages of the *Alaria* genus. Their flukes, known to cause human infections, are found in wild animals inhabiting wetland areas, and these can serve as possible reservoirs [11]. In some countries, the prevalence of *Alaria alata* in raccoon dogs is very high. For example, in Estonia, 68.3% of racoon dogs were infected with this fluke [5]. A common cause of alariosis in humans is consumption of raw or undercooked meat from intermediate hosts such as snails or frogs, or paratenic hosts, such as game [12]. The most common source of infection is wild boar meat, in the diet of which, in special cases, raccoon dogs (carrion) can be found.

Studies conducted in various parts of Europe indicated that racoon dogs should be considered an important source of zoonotic agents, as well as foxes. Sutor et al. [13] suggested that this species should be monitored more intensively in the matters of vector-borne diseases.

The aim of the study was to (1) describe the species composition of the parasitofauna of raccoon dogs from northern Poland, with a particular emphasis on particular species of helminths known to be dangerous to humans, and (2) determinate the prevalence and intensity of infection with noted parasites.

## 2. Materials and Methods

A total of 96 raccoon dogs were obtained from hunters from northern Poland in the period of September 2018 to October 2021. In Poland, the raccoon dog is regarded as an invasive species and is not subject to protection from hunting [1]. In addition, according to Polish law, the collection of tissues and organs from animals killed for non-scientific or non-didactic reasons is not classified as experimental work, and therefore does not require ethics committee approval [14].

Each individual was weighed and measured. Age was determined via tooth wear [15]. Individuals < 1 year were classified as juveniles and individuals > 1 year as adults.

The digestive tract was taken for examination. The intestines were examined using the sedimentation and counting technique (SCT) [16]. Each intestine was opened along its full length and examined macroscopically for large helminths. Then, the intestine was cut into 20 cm long segments and transferred to a glass flask with physiological saline solution (1L). After shaking, the mucosa was stripped between two pressed fingers (the intestine was removed from flask). The fluid with the intestinal material was sedimented several times for 15 min and the supernatant was decanted. The sediment was replaced in small portions to Petri dishes and examined under a stereomicroscope. 

The species composition of the parasitofauna of the studied raccoon dogs was determined by morphological examination (shape, dimensions, structural features) according to the key presented by Khalil et al. [17], Yamaguti [18], Bray et al. [19] and Anderson et al. [20]. The prevalence and intensity of infestation were determined. 

The prevalence of individual species of parasites was compared between juvenile and adult raccoon dogs, as well as between sexes, using the χ^2^ test. The intensity of infection between sexes and between age classes was compared with the Mann–Whitney U test. The confidence interval of a proportion was calculated by the modified Wald method, as recommended by Agresti and Coull [21]. Differences were determined to be statistically significant at *p* < 0.05. All statistical analyses were performed using Statistica 13.0 software (TIBCO Software Inc., Palo Alto, CA, USA).

## 3. Results

Species/genus of tapeworms were identified with consideration of body length, shape of scolex, appearance of rostellar hooks (shape, total length, and blade length of large and small hooks), suction cups and features of the strobila such as the position of genital pore in proglottids and the shape of the uterus. *T. canis* nematodes were identified based on the following features: on body length, appearance of the intestinal cecum and interlabia, the shape of cervical alae, the spicule length and lip structure, etc. *U. stenocephala* was identified based on body length, structure of the buccal capsule and configuration of the lateral bursal rays. *A. alata* was identified with consideration of the body length and width, shape of the body (two sections), and the number and location of the suction cups.

The prevalence and intensity of infestation by raccoon dog parasites is presented according to host age in Table 1, and host sex in Table 2. 

The extensity of infection with all internal parasites was 60.3%. *Echinococcus multilocularis* was observed in 10.42%, *Toxocara canis* in 18.75%, *Alaria alata* in 25.0%, *Teania* spp. in 19.79%, *Uncinaria stenocephala* in 27.08%, *Mesocestoides* spp. in 54.17% and *Dipylidium caninum* in 6.25% of tested racoon dogs. 

No significant (*p* < 0.05) differences in the extensity of infection with particular parasite species were observed between juvenile and adult animals (Table 1) or between males and females (Table 2). 

The mean intensity of infection was shown to be 31 individuals. The highest mean intensity of infection was demonstrated by *A. alata* and *E. multilocularis* then by *Mesocestoides* spp. (Table 1 and Table 2). In the case of *A. alata*, 10 and 22.9 individuals were noted in juvenile and adult animals, respectively, and 20.2 and 21.4 individuals in males and females, respectively. *E. multilocularis* was found only in adults, and in this group of animals mean intensity of infection was 23.3 individuals.

The mean intensity of infection with *Mesocestoides* spp. in juveniles was 16 and in adults—10.2 individuals. In males and females, the mean intensity of infection was 11 and 11.6 individuals, respectively. Statistical analysis did not show that there were significant differences (*p* < 0.05) between the studied groups of animals.

## 4. Discussion

The habitat conditions in Poland are very favourable for raccoon dogs, which are known to inhabit forests and wetlands, as well as the shores of lakes and rivers [22]. 

*Alaria alata* (Goeze, 1782) is a parasitic fluke that it mainly found in the small intestine of canines [11]. However, it may present a danger to humans, who can become paratenic hosts after eating food of animal origin containing mesocercaria, such as undercooked frogs’ legs or raw or semi-raw products derived from wild boar meat. Although alariosis is rare in humans, infection is associated with serious health risks and can be fatal; the risk of severe infection is increased by the fact that the condition has few characteristic symptoms. In the host, the parasites are believed to pass from the intestines to the surrounding tissues, from where they become situated in the liver, kidneys, brain, lungs and adipose tissue [23]. The same authors also propose that alariosis should be classified as an emerging disease, i.e., one of growing importance. 

In the present study, 25.0% of raccoon dogs were found to be infected with *Alaria alata* flukes. This extensity of infection is not excessively high: similar findings were obtained by Duscher et al. [24], who note a 30% prevalence in raccoon dogs from Austria. In contrast, significantly higher prevalence was noted in raccoon dogs and foxes in north-eastern Poland, amounting to 94% and 93%, respectively. The occurrence of *A. alata* has been found to range from 10 to 70% in raccoon dogs depending on the region [25]. 

In Poland, *A. alata* appears to be most common in counties with large percentages of surface water cover [26]. Indeed, in wet areas inhabited by snails and amphibians, the fluke can easily close its development cycle and a high extensity of *Alaria alata* infection can be observed among wild animals acting as its terminal and paratenic hosts. 

Prokopowicz et al. [27] report the incidence of human cases of alariosis in Poland following the consumption of wild boar and goose meat subjected to improper thermal treatment. According to Strokowska et al. [28], *Alaria alata* mesocercariae was identified in44.5% tissue samples of wild boars in north-eastern Poland. The boar is an omnivorous animal, consuming a diet consisting of 80–90% plant matter and 2–11% animal matter. The animal food typically includes earthworms, insects and their larvae, small rodents, eggs and chicks of ground-nesting birds, and frogs. They are also known to eat carrion, including raccoon dogs and foxes [29,30]. As such, the high prevalence of *Alaria alata* in raccoon dogs is a potential threat to human health. 

Along with *Plasmodium falciparum* (malaria), the tapeworm *Echinococcus multilocularis* has been recognized as one of the most dangerous parasites for humans [31]. In humans, the larval forms of the tapeworm are known to be responsible for a disease called alveolar echinococcosis. In Europe, the number of cases ranges from 2 to 40 cases per 100,000 inhabitants in endemic areas [32]. In Poland, *Echinococcus multilocularis* and *E. granulosus* were found in 109 patients during the period 2009 to 2012 (data from Państwowego Zakładu Higieny—National Institute of Hygiene). In 2019 one fatal case due to infection by *E. multilocularis* was reported in the EU (in Poland); thus, the mortality from this zoonotic disease in the EU has been estimated at 0.86% [8].

In the present study, 10.42% of tested raccoon dogs were infected with *Echinococcus multilocularis*, which may suggest that raccoon dogs, next to foxes, are an important reservoir of this parasite for humans. Similar results were obtained by Machnicka et al. [33], who identified *E. multilocularis* in 8% of raccoon dogs from northern Poland, and by Schwarz et al. [34] who report a prevalence of 6% to 12% in raccoon dogs in Brandenburg, eastern Germany. A significant increase in the prevalence of *E. multilocularis*, from 5.10% in 2010 to 15.8% in 2015, was also noted in raccoon dogs in Lithuania, with a mean extensity of infection of 8.1% [35]. Recent years have also seen an increase in the extensity of infection in endemic areas in Europe in foxes [36]. Karamon et al. [37] reported that prevalence of *E. multilocularis* in red fox from different parts of Poland was 25.6% and mean intensity of infection 4473.8.

The distribution of this parasite in Poland is uneven. The highest prevalence has been observed in the Warmińsko-Mazurskie (NE Poland) and Podkarpackie (SE Poland) voivodships, more specifically, 62.9% around Olecko-Gołdap, 41.7% around Ełk, 46.2% around Pisz and 55.6% around Ostrów Mazowiecki. Recently, a rapid increase in *E. multilocularis* infection has been observed in foxes. This high level of infection has been attributed to the large number of eggs excreted with their faeces, the high density of foxes and the significant resistance of eggs to the external environment [38,39,40]. In raccoon dogs, the prevalence of *E. multilocularis* is probably limited by their low population density and the fact that they are less likely to eat rodents compared to foxes [41]. However, this situation may change as the number of raccoon dogs grows.

In Poland, screening studies in humans in selected endemic regions of the Warmińsko-Mazurskie and Podkarpackie voivodships identified a relationship between the prevalence of tapeworms and that of multichamber echinococcosis in humans, this being the highest in the country [6]. In addition, the area with the highest prevalence of *E. multilocularis* in foxes (Warmińsko-Mazurskie voivodship) was also the area with the highest number of cases of alveococcosis among the population. In humans, the disease symptoms resemble those seen in the course of neoplastic disease. The most frequently affected organ is the liver, which displays numbers of small parasite cysts, infiltration by lymphocytes, histiocytes and granulocytes, as well as necrotic foci. Lamellar larval layers grow into healthy tissues from the area around the primary site of invasion, and these may metastasize to other organs [42,43]. Although the main final host responsible for the spread of *Echinococcuss multilocularis* eggs in Poland is the fox, the threat posed by the raccoon dog should also be taken into account.

Toxocarosis is a parasitic zoonotic disease in humans caused by infection with eggs containing third-stage (L3) larvae of *Toxocara canis* and *T. cati*. Humans can become infected by *Toxocara* spp. from foxes and raccoon dogs due to non-compliance with hygiene rules, for example, by failing to keep hands clean or after eating undergrowth contaminated with parasite eggs. Humans are random hosts, in whom the parasites remain in their larval stage after infection. They are situated in various organs and tissues; however, they are most often observed in the liver, and to a lesser extent in the lungs, eyeballs and brain [44,45,46]. 

In the present study, the prevalence of *Toxocara canis* infection in raccoon dogs was found to be 18.75%. Similar results were obtained by Karmon et al. [47], who report a prevalence of 15.1% in raccoon dogs in north-eastern Poland, and a lower value (13.1%) in Denmark [48]. These findings suggest that infected raccoon dogs do contribute to environmental contamination with *Toxocara canis* eggs. This situation poses a threat to human health.

It has been proposed that toxocarosis may be the most common helminthozoonosis in developed countries [49]. The seroprevalence of anti-*T. canis* antibodies in humans has been estimated to range from 1–6% in Japan, 2–4% in Denmark, 6% in Austria, 7% in Sweden, 14% in the USA, 22% in Iran, and 81% in Nepal [49]. In Europe, 1–4% of positive serological reactions are found in adults [50]. However, studies performed 12 years ago in Poland found an increase in the number of cases of toxocariasis among children and adolescents up to 18 years of age, with extensities ranging from 20.7% to 46.4% being observed in the Podlaskie, Mazowieckie and Łódzkie voivodships [51].

In the present study, no statistically significant differences in the occurrence of parasites were found with regard to sex or age of the host. Similar findings were obtained by Bagrade et al. [35] in Lithuania. 

## 5. Conclusions

The prevalence of parasite infection in raccoon dogs living in northern Poland has significantly increased in recent years, and the species composition of the parasitofauna has changed significantly. Like the fox, the raccoon dog may be one of the main final hosts of *Echinococcus multilocularis* and infection may increase the risk of zoonosis in humans. Furthermore, the studied raccoon dogs were found to harbour *Alaria alata*, and this may also pose a threat to human health: the parasite is resistant to freezing and high temperatures. 

Therefore, there is a need to monitor the degree of infection by gastrointestinal parasites in raccoon dogs, especially parasites that can be a source of human infection to fully assess the epizootic situation. The data presented herein indicate that the raccoon dog population is a significant reservoir of *Toxocara* spp. for humans, and the continual increase in density and spread of raccoon dogs may result in a greater risk of infection to humans.

## Figures and Tables

**Table 1:** Prevalence of gastrointestinal parasites in raccoon dogs with regard to host age (J—juvenile, A—adult).

Parasite	Age Group	Number of Infected/Tested	Prevalence (%) (95% CI)	χ^2^ Test Value	Intensity of Infection				
					Mean	GM	Median	Range	Mann–Whitney U Test Value
*Echinococcus multilocularis*	J	0/18	0.0 (0.0–0.2)	χ^2^ = 2.58 *p* = 0.11	0	0	0	--	--
	A	10/78	12.8 (6.9–22.2)		23.3	20.4	23	8–41	
*Toxocara canis*	J	6/18	33.3 (16.1–56.4)	χ^2^ = 3.09 *p* = 0.08	11.5	9.3	13	2–20	U = 21.5 *p* = 0.18
	A	12/78	15.4 (8.9–25.2)		8.8	8.5	9	6–12	
*Alaria alata*	J	4/18	22.2 (8.5–45.6)	χ^2^ = 0.09 *p* = 0.76	10.0	8.6	8	5–20	Z = 1.86 *p* = 0.06
	A	20/78	25.6 (17.2–36.4)		22.9	18.4	17	6–62	
*Taenia* spp.	J	6/18	33.3 (16.1–56.4)	χ^2^ = 2.56 *p* = 0.11	2.7	2.1	2	1–6	U = 35.0 *p* = 0.77
	A	13/78	16.7 (9.9–26.6)		3.2	2.4	2	1–8	
*Uncinaria stenocephala*	J	5/18	27.8 (12.2–51.2)	χ^2^ = 0.01 *p* = 0.94	5.8	3.2	2	1–20	Z = 1.12 *p* = 0.26
	A	21/78	26.9 (18.3–37.7)		7.4	5.4	4	1–20	
*Mesocestoides* spp.	J	10/18	55.6 (33.7–75.5)	χ^2^ = 0.02 *p* = 0.90	16.0	9.5	13	1–41	Z = 1.49 *p* = 0.15
	A	42/78	53.9 (42.9–64.5)		10.2	4.8	9	1–41	
*Dipylidium caninum*	J	0/18	0.0 (0.0–0.2)	χ^2^ = 1.48 *p* = 0.22	0	0	0	--	--
	A	6/78	7.7 (3.3–16.1)		3.0	2.6	3	1–5	
All	J	10/18	55.6 (33.7–75.5)	χ^2^ = 0.14 *p* = 0.71	31.4	26.8	34	8–62	Z = 0.59 *p* = 0.55
	A	47/78	60.3 (49.2–70.4)		30.6	19.4	29	1–133	

**Table 2:** Prevalence of gastrointestinal parasites in raccoon dogs with regard to host sex (m—male, f—female).

Parasite	Sex	Number of Infected/Tested	Prevalence (%) (95% CI)	χ^2^ Test Value	Intensity of Infection				
					Mean	GM	Median	Range	Mann–Whitney U Test Value
*Echinococcus multilocularis*	m	5/48	10.4 (4.1–22.6)	χ^2^ = 0.00 *p* = 1.00	21.4	20.3	17	14–31	U = 10.0 *p* = 0.69
	f	5/48	10.4 (4.1–22.6)		25.2	20.6	33	8–41	
*Toxocara canis*	m	11/48	22.9 (13.2–36.7)	χ^2^ = 1.09 *p* = 0.30	9.9	8.6	10	2–20	U = 37.5 *p* = 0.93
	f	7/48	14.6 (6.9–27.5)		9.3	9.1	9	6–12	
*Alaria alata*	m	12/48	25.0 (14.8–38.9)	χ^2^ = 0.00 *p* = 1.00	20.2	16.7	19	5–45	Z = 0.38 *p* = 0.71
	f	12/48	25.0 (14.8–38.9)		21.4	15.8	13	6–62	
*Taenia* spp.	m	11/48	22.9 (13.2–36.7)	χ^2^ = 0.59 *p* = 0.44	3.8	2.8	2	1–8	U = 30.5 *p* = 0.27
	f	8/48	16.7 (8.4–29.8)		2.0	1.8	2	1–4	
*Uncinaria stenocephala*	m	14/48	29.2 (18.2–43.3)	χ^2^ = 0.21 *p* = 0.65	7.4	5.6	4	2–20	Z = 0.68 *p* = 0.49
	f	12/48	25.0 (14.8–38.9)		6.7	4.2	4	1–20	
*Mesocestoides* spp.	m	30/48	62.5 (48.3–74.8)	χ^2^ = 2.69 *p* = 0.10	11.0	5.5	10	1–41	Z = 0.98 *p* = 0.98
	f	22/48	45.8 (32.6–59.7)		11.6	5.4	9	1–41	
*Dipylidium caninum*	m	4/48	8.3 (2.8–20.1)	χ^2^ = 0.71 *p* = 0.40	3.0	2.5	3	1–5	U = 4.0 *p* = 1.00
	f	2/48	4.2 (0.4–14.8)		3.0	2.8	3	2–4	
All	m	33/48	68.8 (54.6–80.1)	χ^2^ = 3.50 *p* = 0.06	28.7	18.8	31	1–71	Z = 0.07 *p* = 0.94
	f	24/48	50.0 (36.4–64.8)		33.6	23.4	27	1–133	

## Data Availability

The data presented in this study are available on request from the corresponding author.
